# Arthroscopic Assessment of Stifle Synovitis in Dogs with Cranial Cruciate Ligament Rupture

**DOI:** 10.1371/journal.pone.0097329

**Published:** 2014-06-03

**Authors:** Jeffrey P. Little, Jason A. Bleedorn, Brian J. Sutherland, Ruth Sullivan, Vicki L. Kalscheur, Megan A. Ramaker, Susan L. Schaefer, Zhengling Hao, Peter Muir

**Affiliations:** 1 Comparative Orthopaedic Research Laboratory, School of Veterinary Medicine, University of Wisconsin-Madison, Madison, Wisconsin, United States of America; 2 Research Animal Resources Center, Comparative Pathology Laboratory, University of Wisconsin-Madison, Madison, Wisconsin, United States of America; University of Missouri, United States of America

## Abstract

Cranial cruciate ligament rupture (CR) is a degenerative condition in dogs that typically has a non-contact mechanism. Subsequent contralateral rupture often develops in dogs with unilateral CR. Synovitis severity is an important factor that promotes ligament degradation. Consequently, we wished to evaluate the utility of arthroscopy for assessment of stifle synovitis in dogs with CR. Herein, we report results of a prospective study of 27 dogs with unilateral CR and bilateral radiographic osteoarthritis. Arthroscopic images and synovial biopsies from the lateral and medial joint pouches were obtained bilaterally and graded for synovial hypertrophy, vascularity, and synovitis. Synovial tartrate-resistant acid phosphatase-positive (TRAP^+^) macrophages, CD3^+^ T lymphocytes, Factor VIII^+^ blood vessels, and synovial intima thickness were quantified histologically and related to arthroscopic observations. Risk of subsequent contralateral CR was examined using survival analysis. We found that arthroscopic scores were increased in the index stifle, compared with the contralateral stifle (*p*<0.05). Numbers of CD3^+^ T lymphocytes (S_R_ = 0.50, *p*<0.05) and TRAP^+^ cells in joint pouches (S_R_ = 0.59, *p*<0.01) were correlated between joint pairs. Arthroscopic grading of vascularity and synovitis was correlated with number density of Factor VIII^+^ vessels (S_R_>0.34, *p*<0.05). Arthroscopic grading of villus hypertrophy correlated with numbers of CD3^+^ T lymphocytes (S_R_ = 0.34, *p*<0.05). Synovial intima thickness was correlated with arthroscopic hypertrophy, vascularity, and synovitis (S_R_>0.31, *p*<0.05). Strong intra-observer and moderate inter-observer agreement for arthroscopic scoring was found. Dog age and arthroscopic vascularity significantly influenced risk of contralateral CR over time. We conclude that arthroscopic grading of synovitis is a precise tool that correlates with histologic synovitis. Arthroscopy is useful for assessment of stifle synovitis in client-owned dogs, and could be used in longitudinal clinical trials to monitor synovial responses to disease-modifying therapy.

## Introduction

Non-contact cranial cruciate ligament rupture (CR) causes approximately 20% of canine lameness and results in substantial healthcare costs [1]. Residual lameness is common in affected dogs, even after surgical stabilization [2]. Validation of arthroscopic assessment of stifle synovitis in dogs has not been previously described. Synovitis is not routinely assessed during clinical treatment [3]. Consequently, the clinical value of arthroscopic assessment of stifle synovium for evaluation of disease severity in affected dogs is unclear. Recent research suggests that synovitis, assessed using arthroscopy and histologic examination, is a key early event in the explanatory mechanism for CR disease progression [4]–[6].

Lymphoplasmacytic synovitis is typically found in the contralateral stable stifle of dogs with unilateral CR in association with fraying of the cranial cruciate ligament [4],[5],[7],[8]. The inflammatory cell population is principally mononuclear and includes T and B lymphocytes, activated tartrate-resistant acid phosphatase-positive (TRAP^+^) macrophages, major histocompatibility complex (MHC) class II^+^ dendritic cells, and plasma cells [9]–[11]. Fraying and degeneration of the caudal cruciate ligament is also common in affected dogs [12]. Experimentally, persistent stifle synovitis induces significant weakening in the tensile strength of the cranial cruciate ligament [13], and also significantly increases the risk of subsequent contralateral rupture in client-owned dogs [5],[6]. Collectively, these observations suggest that progressive fraying of the cruciate ligaments and development of stifle synovitis are important and related components of the mechanism that leads to progressive tearing of cranial cruciate ligament fibers over time. Synovitis is also an important feature of the pathology of osteoarthritis (OA) in human beings [14]–[16]. Consequently, disease-modifying medical therapy has been studied as an OA treatment for human patients [17]. Collectively, these findings argue that stifle synovitis may be a therapeutic target in dogs with incipient CR [18]. Improved diagnostic methods for assessment of synovitis would be advantageous to patient management and clinical trial design. Arthroscopic assessment of the stifle synovium may be a valuable diagnostic tool that could be used to evaluate and stage dogs with stable partial CR.

A quantitative scoring system based on arthroscopic grading is the gold standard for evaluation of articular cartilage and synovium in human patients; arthroscopic evaluation correlates with histologic synovitis [19]–[21]. No similar grading has been performed in dogs. Arthroscopic examination also provides a magnified view of the joint with improved observation of all stifle joint regions and reduced patient morbidity, compared with arthrotomy [19].

Systemic and synovial fluid biomarkers may also reflect the severity of synovitis. Plasma C-reactive protein (CRP), an acute phase protein, is typically elevated in the serum of dogs with inflammatory arthritis [22]. Interferon-γ (IFN-γ) and tumor necrosis factor-α (TNF-α) are important effector cytokines of Th1 lymphocytes and activated macrophages, respectively. These cytokines play a key role in the development and maintenance of synovitis [14] and may reflect severity of synovitis.

Whilst factors that initiate CR and factors that promote disease progression may not be identical, there is a growing body of scientific evidence suggesting that stifle synovitis promotes disease progression [4]–[6],[13],[23]. Because of the high prevalence of bilateral disease at the time of clinical diagnosis, there is growing interest in clinical trial designs that evaluate development of subsequent contralateral rupture in dogs with unilateral complete CR [6]. The purpose of the present study was three-fold. (1) To develop a validated method for arthroscopic assessment of stifle synovitis in dogs. (2) To determine how arthroscopic grading may relate to inflammatory cell populations within the stifle synovium of dogs with CR, and other clinically relevant biomarkers. (3) Establish whether arthroscopic evaluation of synovitis predicts subsequent contralateral CR in dogs with unilateral CR. We hypothesized that severity of stifle inflammation assessed arthroscopically in dogs with CR would correlate with histologic synovitis and predict risk of subsequent contralateral CR. Additionally, we hypothesized that severity of stifle synovitis in dogs with CR would correlate with objective markers of joint inflammation in serum and synovial fluid.

## Materials and Methods

### Dogs

Client-owned dogs (n = 27) presented to University of Wisconsin-Madison UW Veterinary Care Hospital for surgical stabilization of unilateral CR were prospectively enrolled. Inclusion criteria were: clinical signs of lameness in the index pelvic limb, palpable cranial translation of the tibia relative to the femur indicative of unstable CR, no evidence of contralateral stifle joint instability based on joint palpation under sedation, and radiographic evidence of bilateral OA. Dogs were excluded if data from the history and physical exam suggested a traumatic injury, if other stifle pathology such as patellar luxation was present, or if previous stifle surgery had been performed. Age, weight, gender, and previous pertinent history were recorded for each dog. Passive stifle stability was assessed with cranial drawer and cranial tibial thrust tests performed by a single observer (JPL) under butorphanol (0.2 mg/kg) and dexmedetomidine (4 µg/kg) sedation. Stifle stability was defined as the absence of cranial tibial translation in both tests [24].

#### Group 1 (n = 12)

In this group, dogs were excluded if medications other than non-steroidal anti-inflammatory drugs had been administered in the previous 30 days. Arthroscopic examination of both stifles was performed (see below).

#### Group 2 (n = 15)

In this group, dogs received the same arthroscopic examination as Group 1. Inclusion criteria were availability of clinical follow-up for at least 1 year and arthroscopic images of sufficient quality to enable arthroscopic grading as detailed below. Immediately after surgery, 20 mg of hyaluronic acid (Hylartin V, Pfizer Animal Health, New York, NY) was injected into each stifle and doxycycline was given orally at 5 mg/kg b.i.d. for 10 weeks. Clinical, radiographic, and arthroscopic findings and preliminary survival analysis in this original cohort of 16 dogs have been previously reported [4],[23]. One dog was excluded (see below).

### Ethics Statement

All procedures at the University of Wisconsin-Madison were conducted with the approval of the Animal Care & Use Committee, School of Veterinary Medicine, University of Wisconsin-Madison (V1070). Dogs were recruited with informed written consent from each owner.

### Radiography

#### Group 1

Bilateral stifle radiographs were performed in each dog to meet the inclusion criteria for the study. Stifle radiographs were graded for synovial effusion and osteophytosis using a standard method [25]. Preoperative horizontal-beam weight-bearing lateromedial stress radiographs of both stifles were also made before surgery [26]. Functional cranial cruciate ligament length was determined for left and right stifles from these lateral weight-bearing radiographic images [26]. Digital radiographs were calibrated with a 100 mm bar in each projection and measurements were made using standard medical imaging software (E-film, Merge Healthcare, Chicago, IL).

#### Group 2

Standard orthogonal radiographs of each stifle were made as in Group 1 and graded for synovial effusion and osteophytosis as described above.

### Arthroscopy

**Figure 1:**
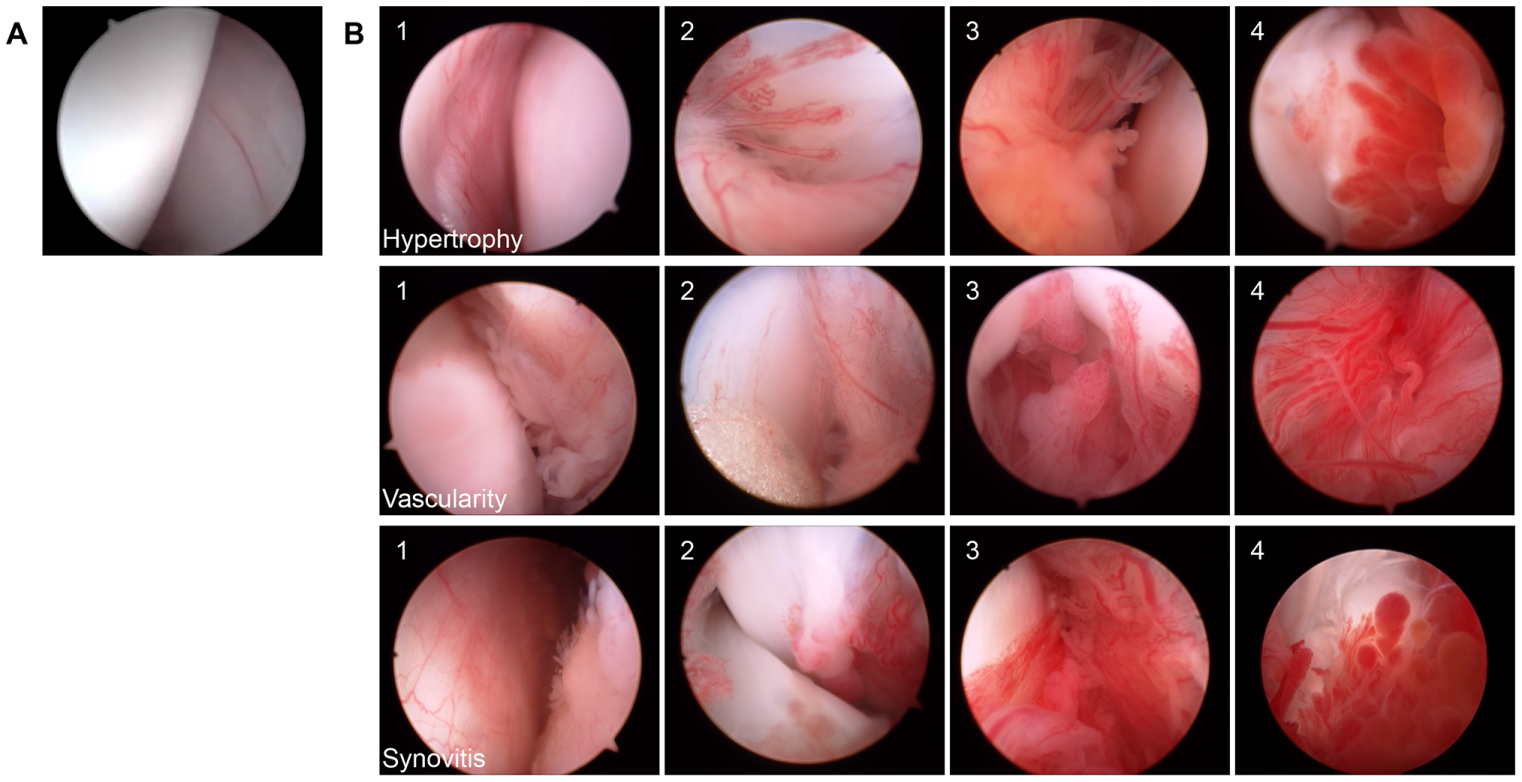
Arthroscopic appearance of stifle synovium in dogs with and without non-contact cruciate rupture. (**A**) Normal stifle synovium of the medial pouch in a healthy young female Hound for reference to ‘B’ [4]. Arthroscopic hypertrophy, vascularity, and synovitis scores of 0. (**B**) Examples of arthroscopic hypertrophy, vascularity, and synovitis scoring in various regions of the canine stifle joint. Arthroscopic scoring ranged from 1–4 [27].

A medial parapatellar mini-arthrotomy was performed on each stifle joint. Each joint was systematically examined using a 2.7 or 2.9 mm 30° rigid arthroscope (Linvatec, Largo, FL) placed in the mini-arthrotomy incision. Stifle joint regions (lateral and medial pouches, lateral and medial femoro-tibial joint compartments, the intercondylar notch, and the femoro-patellar joint) were evaluated arthroscopically. Photographs and digital video were collected for blinded evaluation by three independent observers: a Diplomate of the American College of Veterinary Surgeons (JAB), a small animal surgery resident in the second year of training (JPL), and a first year small animal surgery resident (BJS). Before arthroscopic scoring, each observer completed a tutorial containing calibrating images by af Klint et al. (2009) to improve consistency of arthroscopic assessment among observers. (See http://www.biomedcentral.com/content/supplementary/ar2714-S2.ppt). Disease severity was graded arthroscopically using a scoring system used to assess human knee synovitis (Appendix S1) [27]. Three parameters describing macroscopic inflammation were evaluated for each joint region: Synovial hypertrophy, vascularity, and synovitis (**Fig. 1**). The hypertrophy score graded the visible synovial mass from 0–4 with Grade (0) representing a thin synovial membrane, with only occasional small transparent villi; Grade (1) representing minor thickening; Grade (2) representing granulations; Grade (3) representing abundant villous projections; and Grade (4) representing large, club-like villi. The vascularity score assessed density, size, and tortuosity of vessels with Grade (0) representing thin scattered vessels, progressing to large, abundant vasculature for a maximum score of Grade (4). Finally, the synovitis score evaluated total joint inflammation with Grade (0) representing no active inflammation and Grade (4) representing the most inflamed tissue. Additionally, global synovitis severity was scored using a visual analog scale (VAS) (0–100) for each stifle joint, with 0 representing no inflammation, and 100 signifying the most severe inflammation. The cranial and caudal cruciate ligaments were inspected and probed for evidence of pathologic change, including fiber fraying and the presence of deeper splits within the bulk of each ligament. Fiber disruption was estimated with a calibrated arthroscopic probe. The lateral and medial menisci were inspected and probed for tears. In Group 1 dogs, representative biopsies of synovial membrane were then collected from the medial and lateral joint pouches using a #64 beaver blade.

### Histology

Immediately after collection, biopsies were fixed in Zamboni's fixative for 1–2 days at 4°C [28]. Multiple paraffin-embedded sections, 10 µm thick, were prepared from each tissue block. Sections were stained with hematoxylin and eosin stain for assessment of cellular infiltration. Additional slides were deparaffinized for immunohistochemical staining. Factor VIII staining was based on an established method [29].

For Factor VIII immunohistochemical staining, slides were treated with 0.05% pronase in phosphate buffered saline (PBS) for 10 minutes at 37°C and 0.01% trypsin in 0.1% calcium chloride for 20 minutes at 37°C in a humidified chamber. Sections were cooled to room temperature for 10 minutes and then rinsed in PBS twice for 5 minutes. Sections were treated with 3% H_2_O_2_ in PBS for 10 minutes to block endogenous peroxidase activity and then rinsed in PBS. Sections were blocked with 10% normal goat serum in PBS for 30 minutes to block non-specific binding of secondary immunoglobulins. Primary antibody staining used a polyclonal rabbit anti-human Factor VIII (#A0082 Dako, Carpinteria, CA) that is cross-reactive with the dog, diluted 1∶500 in PBS at room temperature for 1 hour. Sections were then rinsed in PBS three times for 5 minutes each. Secondary antibody staining used was a biotinylated goat anti-rabbit IgG (#BA-1000, Vector, Burlingame, CA) with HRP-streptavidin (#434323, Invitrogen, San Francisco, CA) reagent diluted 1∶500 in PBS for 30 minutes each at room temperature. Positive staining was identified using 3-amino-9-ethylcarbazole as the chromogen (#00-1111 Invitrogen, San Francisco, CA) and the counterstain was Mayer's hematoxylin. Negative controls were prepared without the primary antibody or without the secondary antibody to validate the assay.

For CD3 immunohistochemical staining, slides were treated with 10 mMol/L sodium citrate buffer (pH 6.0) at 95–100°C for 30 minutes, cooled to room temperature, and then rinsed in PBS twice for 5 minutes. Sections were then treated with 3% H_2_O_2_ in PBS for 10 minutes to block endogenous peroxidase activity and rinsed in PBS. Sections were blocked with 10% normal goat serum in PBS for 30 minutes to block non-specific binding of secondary immunoglobulins. Primary antibody staining used a rat anti-human immunoglobulin (#MCA1477T, Serotec, Raleigh, NC) that is cross-reactive with the dog, diluted 1∶500 in PBS overnight at 4°C. Sections were then rinsed in PBS three times for 5 minutes each. Secondary antibody staining used a biotinylated goat anti-rat IgG (#BA-9400, Vector, Burlingame, CA) with HRP-streptavidin reagent (#434323, Invitrogen, San Francisco, CA) diluted 1∶500 in PBS for 30 minutes each at room temperature. The chromagen and counterstain were identical to Factor VIII staining. Positive controls were performed on sections of dog lymph node. Again, negative controls were prepared without the primary antibody or without the secondary antibody.

Histochemical staining of TRAP was based on an established protocol [30]. A solution of naphthol AS-BI phosphate was prepared by dissolving 25 mg of naphthol AS-BI phosphate in 2.5 ml of n,ndimethylformamide to which was added 45 ml of 0.05 M Trismaleate buffer (pH 5.0). A solution of hexazotized pararosanaline was prepared by dissolving 0.25 g of pararosaniline hydroxychloride in 5 mL of distilled water, to which was added 1.25 mL of hydroxychloric acid. This solution was mixed with an equal volume of 4% sodium nitrite immediately before use. The final reaction mixture for histochemical staining was prepared by adding 4 ml of hexazotized pararosanaline solution to the naphthol AS-BI phosphate solution, together with 50 mM sodium-potassium tartrate. The final reaction mixture was filtered before use. Sections were incubated in the reaction mixture at 37°C for 1 to 2 hours, rinsed in distilled water, counterstained in Mayer's hematoxylin, and mounted. Negative controls were performed without the naphthol AS-BI phosphate to validate the protocol.

### Synovial histomorphometry

Morphometric methods were based on previous work [31]. Biopsy samples were blinded and evaluated by a single observer (JPL). Synovial lining cell layer thickness, seen at the center of five random high power fields, was measured to determine lining layer depth of each biopsy sample [31]. In past work, no improvement in reliability was seen for morphometric assessment of vascular density evaluating 8 or 17 random high-power fields [31]. In a pilot analysis, we determined that 12 random high-power fields reasonably estimated vascularity in each section, as the running mean determined from measurement of multiple fields become stable at n = 12 fields. Therefore, vascular density was determined by counting the number of positive vessels in 12 randomly selected high power fields from the same specimens to yield a vascular number density per mm^2^. Measurements of T lymphocyte number density and activated macrophage number density were made by counting the number of positive cells in the entire tissue area of each biopsy specimen. Tissue area was determined using computerized image analysis (Image J, NIH). The number densities of CD3^+^ T lymphocytes and TRAP^+^ macrophages were then determined per mm^2^. Each aggregate of >10 CD3^+^ T lymphocytes was also recorded per mm^2^.

Hematoxylin and eosin stained sections from each biopsy site were evaluated in a blinded fashion by a board-certified veterinary pathologist (RS). Plasmacytic and lymphocytic inflammatory infiltrates were separately graded on a 0–3 scale. Grade (0) represented minimal or absent inflammatory cells at 10x magnification; Grade (1) represented occasional nodular aggregates of leukocytes or mild diffuse inflammatory infiltrates that did not obscure tissue architecture; Grade (2) represented moderately numerous nodular aggregates of leukocytes or moderate diffuse inflammatory infiltrates that did not obscure tissue architecture; and Grade (3) represented numerous nodular aggregates of leukocytes involving more than 50% of the tissue that obscured tissue architecture, or severe diffuse inflammatory infiltrates.

### Serum and synovial fluid ELISA

In Group 1, serum was collected from each dog before surgery. Synovial fluid was collected from each stifle by arthrocentesis before mini-arthrotomy. Samples were centrifuged within 30–120 minutes of collection and then frozen at −80°C for analysis. Concentrations of C-reactive protein (CRP), interferon-γ (INFγ), and tumor necrosis factor-α (TNFα) were measured in serum and synovial fluid using commercial canine-reactive ELISA assays (CRP -Life Diagnostics Inc., West Chester, PA; INF γ and TNF α R&D Systems, Minneapolis, MN).

### Clinical Follow-up

Re-examination or telephone follow-up with the owner was obtained for all dogs at least one year after surgery. All twelve Group 1 dogs were available for follow-up. In Group 2, of the original cohort of 16 dogs, one dog was excluded because of poor arthroscopic images. Overall, 19 dogs were re-examined after surgery, and a telephone interview was conducted for the remaining 8 dogs (n = 27 for clinical follow-up).

### Statistical analysis

Group 1 data were examined for normality using the Kolmogorov-Smirnov test and reported as mean ± standard deviation (SD) or median (range). Regional variation in dependent variables was determined using repeated-measures one-way ANOVA or the Friedman ANOVA, as appropriate. Post-hoc testing was performed with the Tukey's or Wilcoxon tests, respectively. Radiographic scoring of synovial effusion and osteophytosis in stifle pairs was analyzed using the Wilcoxon matched pairs test. Functional cruciate length in left and right stifles was analyzed using a paired Student's *t* test. The effects of meniscal damage and non-steroidal anti-inflammatory drug (NSAID) usage on dependent variables were examined using the Student's *t* or Mann-Whitney U tests, as appropriate. Relationships between arthroscopic and histologic assessment of synovitis and between limb pairs were examined using the Pearson or Spearman rank order correlations, as appropriate. Correlations between stifles for radiographic findings, arthroscopic data, and serum biochemical markers were analyzed without pooling of regional data (n = 12). Correlations between stifles for histologic data were analyzed with pooling of medial and lateral data (n = 24). Combined arthroscopic or histologic data from each stifle were correlated with pooled radiographic osteoarthritis score or synovial biochemical markers from each stifle (n = 24). Because of a lack of regional differences, correlations between arthroscopic scoring and synovial histology were analyzed by pooling of data from both the lateral or medial joint regions of each stifle (n = 48).

Using Group 1 data, precision of arthroscopic scoring was also determined. One observer (JPL) evaluated all arthroscopic images three times in a blinded fashion to determine intra-observer repeatability of the scoring system using the intraclass correlation coefficient (ICC) statistic. Two other observers (JAB, BJS) also subjectively assessed all arthroscopic images in a blinded fashion. Collectively, these observations were used to determine inter-observer reproducibility of the scoring system using the ICC. ICC≤0.3 were considered weak, coefficients >0.3 and <0.75 were considered moderate, and ≥0.75 were considered strong.

Data from clinical follow-up of Group 1 and Group 2 cases were used for survival analysis (n = 27). The Cox's Proportional Hazards model, the Kaplan-Meier estimator, and logistic regression were used to investigate which clinical factors might influence risk of subsequent contralateral CR. Initially, putative risk factors were analyzed in a univariate model. Factors considered in the univariate model included age, gender, bodyweight, radiographic scoring of synovial effusion and osteophytosis in the index and contralateral stifles, arthroscopic scoring of synovial hypertrophy, vascularity, and synovitis in the index and contralateral stifle, the proportion of the contralateral cranial cruciate ligament that was estimated to be damaged arthroscopically, and use of doxycycline after surgical treatment. Univariate parameters with *p*<0.2 were then considered further in a multivariate model. All results were considered significant at *p*≤0.05.

## Results

### Dogs

#### Group 1

Twelve dogs were prospectively enrolled into the study. The mean ± SD age was 4.8±2.2 years and the mean ± SD weight was 40±11.7 kg. There were five castrated males, one intact male, five spayed females, and one intact female. Mean ± SD time from initial lameness to presentation was 10±7 weeks. There were four Labrador retrievers with the other dogs representing eight different medium to large breeds including Border Collie, Chesapeake Bay Retriever, German Shepherd dog, Great Pyrenees, Rhodesian Ridgeback, Newfoundland, Weimaraner, and one mixed breed dog. Five dogs were given NSAID medication during the 30 days before arthroscopy and five dogs had no history of NSAID treatment. The NSAID status of two dogs was not known. No other medications had been administered to any dog within the preceding 30 days.

#### Group 2

Fifteen dogs were prospectively enrolled in and completed the study. The mean ± SD age was 4.7±2.4 years and the mean ± SD weight was 41.7±11.8 kg. There were six castrated males, one intact male, and eight spayed females. There were three Golden retrievers and two Labrador retrievers with the other dogs representing nine different medium to large breeds including Siberian Husky, Mastiff, Great Dane, Bulldog, Bloodhound, Saint Bernard, German Wirehaired Pointer, Chesapeake Bay Retriever and two mixed breed dogs.

### Radiography

#### Group 1

**Figure 2:**
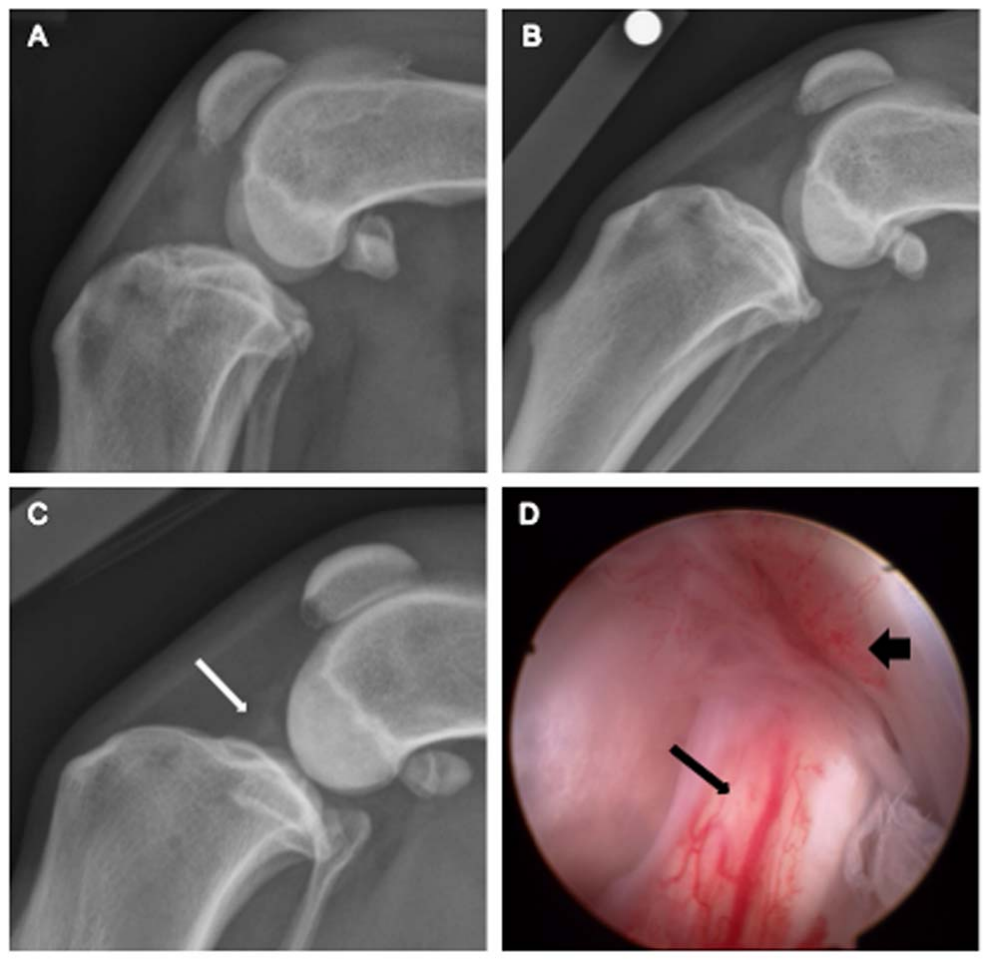
Lateral radiographic images of the stifle (A–C). (**A**) Index stifle with marked osteophytosis and effusion. (**B**) Contralateral stifle with mild osteophytosis and moderate effusion. (**C**) Contralateral stifle with no radiographic osteophytosis and mild effusion (white arrow). (**D**) Same contralateral stifle from image ‘C’ with minimal radiographic changes demonstrating obvious synovitis and fraying of the cranial cruciate ligament (narrow black arrow) and inflammation of the synovium overlying the caudal cruciate ligament (broad black arrowhead).

Evaluation of radiographs of both stifle joints from all dogs revealed synovial effusion. Stifle osteophytosis was variable between dogs (**Fig. 2**). Median (range) score for synovial effusion was 2 (1, 2) for the index stifle and was significantly greater than for the contralateral stifle 1 (1, 2) (*p*<0.05). Median total OA score was 6 (3, 10) for the index stifle was increased compared with the contralateral stifle 2.5 (1, 6) (*p*<0.01). There was no significant correlation in total OA score between the index and contralateral stifles. Mean ± SD functional cruciate length of the index and contralateral stifles were 36.4±5.0 mm, and 30.9±3.5 mm, respectively. Functional cruciate length was increased in the index stifle, when compared with the contralateral stifle (*p*<0.001).

### Arthroscopy

#### Group 1

Synovitis was observed in all stifles. Complete CR was confirmed arthroscopically in all index stifles. Medial meniscal tears were found in 7 of 12 (58%) index stifles. Caudal cruciate ligament fraying was also found in 7 of 12 index stifles (58%). Visible fraying of the cranial cruciate ligament was found in all contralateral stifles. Slight caudal cruciate ligament fraying was found in 2 of 12 contralateral stifles. Meniscal tears were not identified in any contralateral stifle. Results of arthroscopic scoring are summarized in **Table 1**.

**Table 1:** Arthroscopic scoring of synovium in the unstable index stifle and stable contralateral stifle of dogs with non-contact cruciate rupture.

Region	Index Stifle	Contralateral Stifle
*Arthroscopic Hypertrophy*		
Lateral Pouch	*^,a^3.0 (2, 4)	*^,a^2.0 (1, 3)
Lateral Femoro-tibial	^a^3.0 (1, 4)	^a^2.0 (1, 4)
Medial Pouch	^a^3.0 (1, 4)	^a^2.0 (1, 4)
Medial Femoro-tibial	*^a^3.0 (2, 4)	*^a^2.0 (1, 3)
Intercondylar notch	^a^3.0 (2, 4)	^a^2.0 (0, 4)
Femoro-patellar	*^,a^3.0 (2,4)	*^,a^2.0 (0, 3)
*Arthroscopic Vascularity*		
Lateral Pouch	*^,a^2.5 (1, 4)	*^,a^2.0 (0, 2)
Lateral Femoro-tibial	^a^3.0 (2, 4)	^b^2.0 (1, 3)
Medial Pouch	^a^3.0 (1, 4)	^a,b,c^2.0 (0, 4)
Medial Femoro-tibial	*^,a^3.0 (2, 4)	*^,a,b,c^2.0 (1, 3)
Intercondylar notch	*^,a^3.5 (2, 4)	*^,b,c^2.0 (1, 4)
Femoro-patellar	*^,a^ 3.0 (2, 4)	*^,a,c^1.0 (0, 3)
*Arthroscopic Synovitis*		
Lateral Pouch	*^,a^3.0 (2, 4)	*^,a^2.0 (1, 3)
Lateral Femoro-tibial	^a^3.0 (1, 4)	^a^2.0 (1, 3)
Medial Pouch	^a^3.0 (1, 4)	^a^2.0 (0, 4)
Medial Femoro-tibial	*^,a^3.0 (2, 4)	*^,a^1.0 (0, 3)
Intercondylar notch	*^,a^3.0 (2, 4)	*^,a^2.0 (1, 4)
Femoro-patellar	*^,a^3.0 (2, 4)	*^,a^2.0 (0, 3)

**Note**: *Group 1* dogs, n = 12. Values represent median (range). *within a row, medians differ significantly (*p*<0.05). Within a column, medians with different superscript letters differ significantly (*p*≤0.05).

No significant regional variation of synovial hypertrophy, synovial vascularity, or synovitis was found in the index stifle. Similarly, no significant regional variation for hypertrophy or synovitis was found in the contralateral stifle. However, vascularity score in the lateral femoro-tibial compartment was increased when compared with the femoro-patellar joint (*p* = 0.01) and lateral pouch (*p* = 0.01); intercondylar notch vascularity score was also increased when compared with the lateral pouch (*p*<0.05). Arthroscopic scoring for hypertrophy, vascularity, and synovitis was increased in the index stifle in all joint compartments, when compared with the contralateral stifle (*p*<0.05), except for hypertrophy, vascularity, and synovitis in the medial pouch (*p* = 0.6, *p* = 0.06, and *p* = 0.2, respectively), synovitis in the lateral femoro-tibial compartment (*p* = 0.3, *p* = 0.09, and *p* = 0.08 respectively), and hypertrophy in the intercondylar notch (*p* = 0.1) and lateral femoro-tibial compartment (*p* = 0.26). VAS synovitis score for the index stifle (72±13) was increased, when compared with the contralateral stifle (42±23, *p*<0.005). Significant correlations between arthroscopic scoring in the index stifle and the contralateral stifle were not found. The presence of meniscal tearing or historic use of NSAID medication did not significantly influence arthroscopic scoring.

Intra- and inter-observer variability is summarized in **Table 2**. Intra-observer variability was low, with strong ICC for vascularity, synovitis, and global (VAS) synovitis scoring, and moderate ICC for synovial hypertrophy. Inter-observer variability was moderate, with moderate ICC for all arthroscopic scores. All ICC values were significant at *p*<0.0001.

**Table 2:** Precision of arthroscopic scoring of synovial inflammation.

Observers	Observer A (n = 3)	Observers A–C	Observers A & B	Observers A & C	Observers B & C
Hypertrophy	0.719 (0.65, 0.78)	0.463 (0.364, 0.558)	0.424 (0.28, 0.549)	0.506 (0.374, 0.618)	0.451 (0.311, 0.572)
Vascularity	0.801 (0.748, 0.846)	0.683 (0.608, 0.75)	0.658 (0.555, 0.742)	0.739 (0.654, 0.805)	0.642 (0.535, 0.729)
Synovitis	0.808 (0.756, 0.852)	0.649 (0.569, 0.721)	0.622 (0.511, 0.713)	0.676 (0.577, 0.756)	0.643 (0.537, 0.73)
Global VAS	0.877 (0.776, 0.940)	0.678 (0.477, 0.830)	0.616 (0.297, 0.813)	0.811 (0.616, 0.913)	0.536 (0.184, 0.768)

**Note**: VAS – Visual analogue scale. Observer A [JPL], Observer B [BJS], and Observer C [JAB] assessed the arthroscopic images to determine precision of the scoring system [27]. Observer A was used to determine repeatability. All three observers were used to determine reproducibility. Data represents the intraclass correlation coefficient (95% confidence interval). All ICC values were significant at *p*<0.0001. Data are derived from *Group 1* dogs (n = 12).

### Synovial histology

#### Group 1

Data are summarized in **Table 3**. Synovial morphometric measurements were not significantly influenced by the presence of meniscal damage or historic NSAID usage. The width of the synovial intima was 16.1±3.1 µm and 15.0±3.9 µm in the lateral and medial joint pouches of the index stifle. In the contralateral stifle, these values were 14.7±2.9 µm and 14.5±4.0 µm. Differences between index and contralateral stifles were not significant.

**Table 3:** Synovial histomorphometry of unstable index and stable contralateral stifles from dogs with non-contact cruciate rupture.

Biopsy Site	Synovium intimal width (µm)	Factor VIII^+^ vessels (#/mm^2^)	CD3^+^ T lymphocytes (#/mm^2^)	CD3^+^ T lymphocyte aggregates (#/mm^2^)	TRAP^+^ macrophages (#/mm^2^)
Index Lateral	^a^16.1±3.1	^a^0.28±0.08	^a^1.1 (0.0, 28.4)	^a^0.005 (0.0, 0.153)	^a^1.6 (0.0, 29.4)
Index Medial	^a^15.0±3.9	^a^0.28±0.06	^a^0.8 (0.0, 40.9)	^a^0.002 (0.0, 0.196)	^a^1.4 (0.0, 57.2)
Contralateral Lateral	^a^14.7±2.9	^b^0.15±0.07	^a^0.1 (0.0, 6.9)	^a^0.0 (0.0, 0.050)	^b^0.1 (0.0, 1.6)
Contralateral Medial	^a^14.5±4.0	^b^0.19±0.06	^a^0.4 (0.0, 40.2)	^a^0.0 (0.0, 0.055)	^b^0.1 (0.0, 11.3)

**Note**: Data represents median (range) or mean ± standard deviation. Within a column and lateral or medial joint region, median or mean values with differing superscript letters differ significantly (*p*≤0.05). Data are derived from *Group 1* dogs (n = 12).

**Figure 3:**
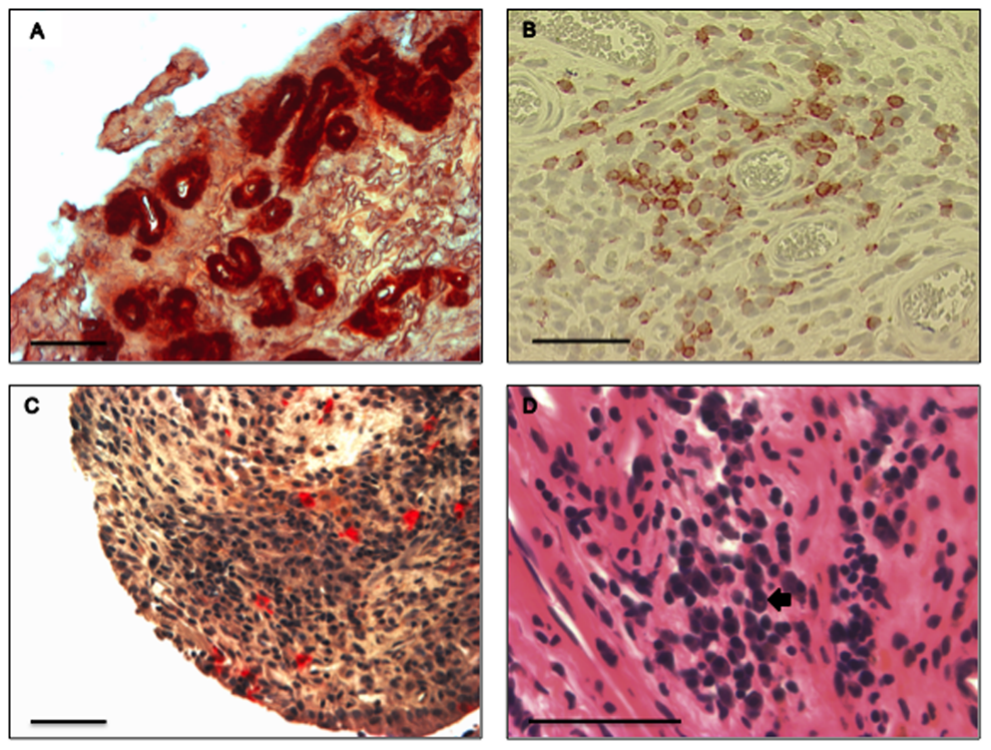
Stifle synovial biopsies from dogs with non-contact cranial cruciate rupture. (**A**) Factor VIII^+^ immunohistochemical staining of blood vessel endothelium. Blood vessel intima stains intensely red. (**B**) CD3 immunohistochemical staining of T lymphocytes. CD3^+^ cells exhibit red staining on the cell surface. Clusters of CD3^+^ T lymphocytes were typically arranged in characteristic aggregates around a blood vessel. (**C**) Histochemical staining for tartrate-resistant acid phosphatase (TRAP) as a marker for activated macrophages. TRAP^+^ macrophages stained intensely red and were often found throughout the synovium in association with other mononuclear cells. (**D**) Hematoxylin and eosin staining typically revealed the presence of a substantial population of mononuclear inflammatory cells within the synovium. Large numbers of plasma cells were typically seen. Plasma cells have a perinuclear clear zone (black arrowhead). A & B - 3-amino-9-ethylcarbazole chromogen, Mayer's hematoxylin counterstain; C - naphthol AS-BI/paraosanaline histochemical stain, Mayer's hematoxylin counterstain; D – hematoxylin & eosin stain. All scale bars are 50 µm.

Vascular density in the synovium was high in all stifle joints (**Fig 3A**). Number density of Factor VIII^+^ blood vessels was increased in both lateral and medial pouches of the index stifle, when compared with the contralateral stifle (*p*<0.001). There were no significant regional differences in the number density of Factor VIII^+^ blood vessels within each joint. Number density of pooled medial and lateral joint pouch Factor VIII^+^ blood vessels in the stifle joint pairs was not correlated.

CD3^+^ lymphocytes were found in 92% of index stifles and 83% of contralateral stifles. Number density of CD3^+^ T lymphocytes in the lateral and medial joint pouches of the index stifle was not significantly different from the contralateral stifle. There were no significant regional differences in CD3^+^ T lymphocytes and number of CD3^+^ aggregates within each joint. The number density of CD3^+^ T lymphocytes was correlated between index and contralateral stifles (S_R_ = 0.50, *p*<0.05, n = 24, **Fig. 3B**), when data from lateral and medial joint pouches in each stifle were pooled.

TRAP^+^ macrophages were found in 83% of index stifles and 67% of contralateral stifles. Number density of TRAP^+^ macrophages in the synovium of the lateral and medial pouches was increased in the index stifle, when compared with the contralateral stifle (*p*<0.05, **Table 3**, **Fig. 3C**). There were no significant regional differences in the number density of TRAP^+^ cells within each stifle joint (**Table 3**). The number density of TRAP^+^ cells was correlated between index and contralateral stifles (S_R_ = 0.59, *p*<0.01, n = 24), when data from lateral and medial joint pouches in each stifle were pooled.

Subjective scoring for lymphocytic and plasmacytic aggregates revealed that the contralateral plasmacytic score was increased in the medial pouch, when compared with the lateral joint pouch (*p*<0.05, **Fig. 3D**). No other significant difference within joints or between joint pairs was evident. The lymphocytic score correlated with the number density of CD3^+^ T lymphocyte aggregates (S_R_ = 0.61, *p*<0.001, n = 24).

### Serum and synovial inflammatory biomarkers

#### Group 1

CRP was detected in the serum of all dogs (27.2±37.3 mg/L). There were no significant differences in synovial CRP concentration between the index (10.6±14.0 mg/L) and contralateral stifles (7.3±7.5 mg/L, *p* = 0.32). Serum and synovial CRP concentrations were significantly correlated for both the index stifle (R^2^ = 0.87, *p*<0.001, n = 12) and the contralateral stifle (R^2^ = 0.65, *p*<0.05, n = 12). Synovial fluid CRP concentrations in the index and contralateral stifles were correlated (R^2^ = 0.66, *p*<0.05, n = 12). The presence of meniscal damage or NSAID usage did not significantly affect CRP concentrations in serum or synovial fluid.

INFγ was detectable in the serum of 3 of 12 dogs (range 22.4–94.3 pg/ml); synovial INFγ was only found in 1 of these 3 dogs. Synovial INFγ was detected in the stifle joints in 4 of 12 dogs (range 129–1,177 pg/ml); INFγ was detected bilaterally in synovial fluid in two of these 4 dogs. TNFα was only detected in the serum of one dog (2.6 pg/ml) and was not detected in synovial fluid in any dog. The presence of meniscal damage or NSAID usage did not significantly affect INFγ concentrations in serum or synovial fluid.

### Radiographic, arthroscopic and histologic correlative analysis

#### Group 1

Data are summarized in **Table 4**. Arthroscopic hypertrophy (S_R_ = 0.35, *p* = 0.01, n = 48), vascularity (S_R_ = 0.31, *p*<0.05, n = 48), and synovitis scores (S_R_ = 0.38, *p* = 0.01, n = 48) were correlated with the width of the synovial intima. Arthroscopic vascularity score (R^2^ = 0.40, *p* = 0.005, n = 48) and synovitis score (S_R_ = 0.34, *p*<0.05, n = 48) were correlated with the number density of Factor VIII^+^ blood vessels. Arthroscopic hypertrophy score was significantly correlated with the number density of CD3^+^ T lymphocyte aggregrates (S_R_ = 0.34, *p*<0.05, n = 48) and subjective scoring for lymphocyte infiltration of the synovium (S_R_ = 0.34, *p*<0.05 n = 48). Arthroscopic hypertrophy (S_R_ = 0.39, *p*<0.01, n = 48), vascularity (S_R_ = 0.32, *p*<0.05, n = 48), and synovitis scores (S_R_ = 0.33, *p*<0.05, n = 48) were also correlated with subjective scoring for plasmacytic infiltration of the synovium. Arthroscopic scoring was not significantly correlated with the number density of TRAP^+^ macrophages. Furthermore, serum or synovial CRP and INFγ concentrations were not significantly correlated with radiographic OA, arthroscopic change, or histologic inflammation of the synovium.

**Table 4:** Relationships between arthroscopic scoring and histological changes in stifle synovium from dogs affected with non-contact cruciate rupture.

	Arthroscopic Hypertrophy	Arthroscopic Vascularity	Arthroscopic Synovitis
Synovial intima thickness	S_R_ = 0.35, *p* = 0.01	S_R_ = 0.31, *p*<0.05	S_R_ = 0.38, *p* = 0.01
Factor VIII^+^ blood vessels	NS	R^2^ = 0.40, *p*<0.01	S_R_ = 0.34, *p*<0.05
CD3^+^ aggregates	S_R_ = 0.34, *p*<0.05	NS	NS
TRAP^+^ macrophages	NS	NS	NS
Plasmacytic inflammation	S_R_ = 0.39, *p*<0.01	S_R_ = 0.32, *p*<0.05	S_R_ = 0.33, *p*<0.05
Serum CRP	NS	NS	NS
Serum interferon γ	NS	NS	NS
Radiographic synovial effusion score	S_R_ = 0.56, *p*<0.001	S_R_ = 0.59, *p*<0.001	S_R_ = 0.60, *p*<0.001
Radiographic osteophytosis score	S_R_ = 0.52, *p*<0.001	S_R_ = 0.45, *p*<0.001	S_R_ = 0.51, *p*<0.001

**Note**: CRP – C-reactive protein. NS - not significant, *p*>0.05. Correlations between arthroscopic and radiographic variables are based on data from *Group 1 and Group 2* dogs (n = 54 stifles from 27 dogs); all other correlations are based on data from *Group 1* dogs (n = 24 stifles from 12 dogs).

Radiographic effusion was correlated with the number density of Factor VIII^+^ vessels (S_R_ = 0.59, *p*<0.005, n = 24), and clusters of CD3^+^ cells (S_R_ = 0.43, *p*<0.05, n = 24). Radiographic osteophytosis was correlated with the number density of Factor VIII^+^ vessels (S_R_ = 0.67, *p*<0.001, n = 24).

#### Groups 1 and 2

Radiographic synovial effusion correlated with arthroscopic hypertrophy score (S_R_ = 0.56, *p*<0.001, n = 54), arthroscopic vascularity score (S_R_ = 0.59, *p*<0.001, n = 54), arthroscopic synovitis score (S_R_ = 0.60, *p*<0.001, n = 54) and with arthroscopic VAS synovitis score (S_R_ = 0.59, *p*<0.001). Radiographic osteophytosis correlated with arthroscopic hypertrophy score (S_R_ = 0.52, *p*<0.001, n = 54), arthroscopic vascularity score (S_R_ = 0.45, *p*<0.001, n = 54), arthroscopic synovitis score (S_R_ = 0.51, *p*<0.001, n = 54) and with arthroscopic VAS synovitis score (S_R_ = 0.49, *p*<0.001).

### Survival Analysis

#### Group 1 and 2

**Figure 4:**
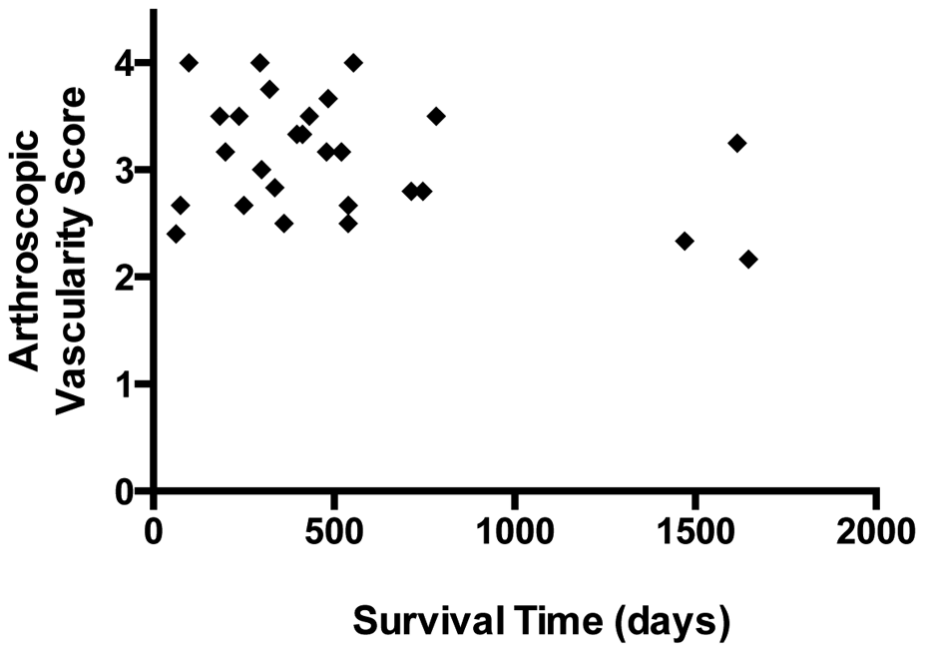
Relationship between arthroscopic vascularity score in the index stifle and contralateral cranial cruciate ligament survival. Cox's Proportional Hazard Ratio is 3.51, *p* = 0.05.

Data from Groups 1 and 2 were pooled for survival analysis (n = 27). Within the study period, 14 of 27 dogs (52%) developed subsequent contralateral rupture. Median time to development of subsequent contralateral rupture was 593 days. The radiographic and arthroscopic findings of Group 2 dogs were similar to the findings in Group 1 dogs. Clinical factors from the univariate model analyses that were considered further in the multivariate model included age, gender, radiographic osteophytosis in the index and contralateral stifles, arthroscopic vascularity in the index stifle, and use of doxycycline after surgery. Regression results from the multivariate model that analyzed all effects from the univariate parameters are reported in **Table 5**. Significant factors were age and arthroscopic vascularity score in the index stifle. Hazard ratio increased with increasing synovial inflammation in the index stifle as assessed by the arthroscopic vascularity score (**Fig. 4**). Every 0.1 increase in the median vascularity score for the index stifle joint compartments was associated with a Hazard Ratio of 1.14. Increasing age was associated with a decrease in Hazard Ratio of 0.57 for every year of age. Other factors in the multivariate model were not significant. However, there was a trend for neutering to also influence contralateral cruciate survival (*p*<0.07, Table 6). On average, shorter survival times were seen in neutered female dogs, when compared with neutered male dogs.

**Table 5:** Multivariate parameter estimates from Cox's Proportional Hazards regression analysis of contralateral cruciate survival.

Parameter	Significance	Hazard Ratio	95% Hazard Ratio Confidence Limits
Age	*p*<0.05	0.57	0.36–0.91
Neutering	NS (*p* = 0.09)	8.38	0.73–96.54
Index stifle radiographic osteophyte score	NS	0.42	0.14–1.22
Contralateral stifle radiographic osteophyte score	NS	0.48	0.17–1.41
Index stifle arthroscopic vascularity score	*p* = 0.05	3.67	1.00–13.52
Postoperative doxycycline treatment	NS	0.75	0.16–3.45

**Note**: NS - not significant, *p*>0.05. Data from *Group 1 and Group 2* dogs (n = 27).

**Table 6:** Effect of gender on median contralateral cranial cruciate ligament survival time.

Group	Median (days)	Range (days)
Female (n = 1)	479	n/a
Ovariohysterectomized Female (n = 13)	362	75–1,470
Male (n = 2)	1007	397–1,616
Castrated Male (n = 11)	484	63–1,647

**Note**: Cox's Proportional Hazard Ratio for the effect of Gender on contralateral cranial cruciate ligament survival is 8.38 at *p* = 0.09.

When logistic regression was used to examine risk factors for development of the contralateral CR by 12 months after diagnosis, clinical factors that were considered further in the multivariate model were gender, radiographic osteophytosis in the index stifle and radiographic effusion in the contralateral stifle. When gender was coded as intact or neutered, neutering was associated with higher risk of contralateral CR. In the final multivariate model, development of subsequent contralateral CR was significantly influenced by gender, osteophytosis in the index stifle (odds ratio [unit change]  = 0.18), and synovial effusion in the contralateral stifle (odds ratio [unit change]  = 7.30) (*p*<0.05).

## Discussion

The present study compared arthroscopic assessment of synovial inflammation to markers of histologic inflammation including synovium thickness, Factor VIII^+^ vessels, CD3^+^ T lymphocytes, TRAP^+^ macrophages, and plasmacytic cellular infiltrate. Additionally, biomarkers of inflammation were quantified and potential relationships with histologic or arthroscopic findings were examined. The arthroscopic synovial assessment method we used was adapted from a validated scoring system used in human beings [27]. Our selection of histologic, serum, and synovial fluid markers was chosen on the basis of past work that has shown that inflamed synovium in CR stifles typically contains a mixed population of mononuclear cells, often aggregated into nodules [10],[11]. We also determined intra- and inter-observer variability to help validate our arthroscopic assessment method. Our results suggest that severity of stifle synovitis assessed arthroscopically in dogs with CR is related to histologic synovitis and reflects the mononuclear inflammatory cell population within stifle synovium. Our results also suggest that arthroscopy is a reliable clinical tool for synovial assessment. However, severity of stifle synovitis in dogs with CR is not significantly related to serum and synovial fluid CRP, INFγ, and TNFα.

To confirm physical examination findings, including the absence of cranial tibial thrust, standing stifle radiographs were made at diagnosis in Group 1 dogs to assess functional cruciate length. This technique has been shown to correlate with CR and associated cranial tibial translation in dogs [26], and is easier to perform than the stress radiography method previously used in our laboratory [4]. All CR stifles demonstrated cranial subluxation of the tibia radiographically, while all contralateral stifles did not have cranial subluxation. Average cranial translation of the tibia in the CR-affected stifles was 6 mm. During arthroscopic examination, we found that all contralateral stifles had some degree of cruciate damage with fiber fraying or splits between bundles of fibers, typically involving less than 10% of the craniomedial band. All of the cranial cruciate ligaments were deemed to be functionally intact in the contralateral joints. Radiographs revealed synovial effusion and osteophytosis in all index stifles with CR at diagnosis. Contralateral stifles usually had mild effusion and osteophytosis; in some dogs only a small degree of effusion was evident in the cranial joint compartment. In contralateral joints with mild radiographic change, arthroscopy often revealed obvious changes in the synovium, suggesting that radiography is not as sensitive as arthroscopic examination for detecting synovitis in the canine stifle [4]. Interestingly, in Group 1 dogs, radiographic effusion, and to a lesser degree osteophytosis, correlated with both arthroscopic and histologic quantification of synovitis. Consequently, subtle radiographic effusion is an early diagnostic marker for CR arthropathy. Radiographic OA in both the unstable index joint and stable contralateral joint has previously been shown to significantly correlate with arthroscopic synovitis [4]. Because contralateral stifles with only minimal effusion radiographically had significant synovitis, it would be interesting to look at another group of contralateral stifles with no radiographic changes to determine the earliest changes in CR affected dogs. It is generally accepted that a proportion of dogs with unilateral cruciate rupture experience a traumatic contact injury, particularly as it is known that avulsion fracture of a cranial cruciate ligament attachment site can occur in dogs [32]. However, the low incidence of avulsion fracture diagnosed in immature dogs and the high prevalence of contralateral pathological change based on radiographic and arthroscopic observation at diagnosis [4] [6] suggests that traumatic contact injury explains only a very small proportion of all canine cruciate rupture cases. Survival analysis provides a useful statistical approach to evaluate risk factors that influence CR disease progression without the need for a cohort of CR-affected dogs with arthroscopically normal contralateral stifles.

Synovitis has been previously defined by intensity, extent, and location [19]. The scoring system we used was comprised of three complimentary categories: hypertrophy, vascularity, and synovitis [27]. Hypertrophy grades the visible synovial mass, but cannot differentiate active from chronic change. Vascularity grades the presence of blood vessels within the synovial villi, and does not account for hyperemia, whereas the synovitis score encompasses hyperemia, which principally reflects active inflammation. Consequently, all three scores can be used for clinical assessment. With this scoring system, the stifle is divided into six joint regions to comprehensively assess what may be a non-uniform disease process. Synovitis may have a variable distribution in joints [27]. Our findings suggest that synovitis in dogs with CR has a uniform macroscopic distribution. No differences were found between the six regions of the stifle for any of the arthroscopic scoring categories in the index stifle. Similarly, we did not find regional differences in the contralateral joint for hypertrophy or synovitis, but did identify some regional differences in vascularity score. The biological explanation for the regional differences we identified in vascularity scores is unclear, but may have resulted from a type I error, since histologic density of Factor VIII^+^ vessels did not demonstrate regional variation in the contralateral stifle.

Arthroscopic evaluation was highly repeatable for a single observer, with moderate repeatability between multiple observers, with varying degrees of arthroscopic experience. One observer (JAB) was a Diplomate of the American College of Veterinary Surgeons with extensive experience of canine stifle arthroscopy, one observer (JPL) was familiar with arthroscopy and in the second year of residency training in small animal surgery, and one observer (BJS) had little experience with arthroscopy and was in the first year of residency training. Because a substantial learning curve with arthroscopy exists, better inter-observer agreement might be obtained if all observers were similarly experienced.

The index stifle with CR was more inflamed in all regions for all arthroscopic scoring categories, although some did not reach significance. This likely reflects low statistical power. VAS synovitis score for the index stifle was significantly higher, compared to the contralateral stifle. The VAS synovitis score was also reliable between and among observers. Interestingly, arthroscopic assessment of synovitis in the stifle joint pairs did not correlate well, in contrast to an earlier study [4]. However, histologic markers of synovitis were correlated between joint pairs in the present study. In accordance with previous work [12], some dogs had arthroscopic evidence of caudal cruciate ligament damage, including two stable contralateral stifles.

The number density of blood vessels [33]–[35], CD3^+^ lymphocytes, and TRAP^+^ macrophages were used to objectively assess histologic inflammation. Vessel number density was increased in the index stifle with CR and correlated well with the arthroscopic vascularity score and the synovitis score, which characterized overall synovitis severity. The width of the synovial intima [31] also correlated well with all arthroscopic scores, including synovial hypertrophy.

Expression of the CD3^+^ epitope was studied, since T-lymphocytes are an important component of the inflammatory cell population in the synovium of dogs with CR [10] [18]. A large majority of index stifles with CR (92%), as well as the contralateral stifles (83%), were positive for synovial CD3^+^ lymphocytes. Synovial CD3^+^ T lymphocytes moderately correlated with the arthroscopic assessment of hypertrophy, and were typically found in aggregates or clusters. These observations reinforce the concept that there is an immune-mediated component to the CR mechanism [18] [36]. An interesting finding was that the number density of CD3^+^ T lymphocytes in the index and stable contralateral joints were significantly correlated, with no significant difference in T lymphocyte numbers between index and contralateral joints. These findings suggest that the inflammatory component to the ligament rupture mechanism starts in the early phase of the condition.

TRAP^+^ mononuclear cells are activated macrophages typically found in synovium of dogs with CR, but not in normal synovium [11]. In the present study, we found that a majority of synovial biopsies from both the index stifle (83%) and the contralateral stifle (67%) contained TRAP^+^ macrophages. Similar to CD3^+^ lymphocytes, numbers of TRAP^+^ macrophages in the stifle pairs were significantly correlated. However, the number density of TRAP^+^ macrophages did not correlate with arthroscopic findings.

Review of the hematoxylin and eosin stained sections of synovium revealed a large lymphoplasmacytic cell population, with a non-uniform distribution in many sections. B-lymphocytes and plasma cells are important components of the inflammatory cell population in the synovium of CR-affected dogs [10]. We found that lymphocyte estimation from these sections moderately correlated with morphometric assessment of CD3^+^ T lymphocyte numbers from sections stained immunohistochemically. This suggests that scoring for plasmacytic inflammation likely has similar reliability. Scoring for plasmacytic inflammation correlated moderately well with all arthroscopic categories.

Histomorphometric findings demonstrated correlations between joint pairs, suggesting a systemic disease mechanism [4]. To further address this concept, we also measured CRP, TNFα, and INFγ in serum and synovial fluid. No significant correlations between the arthroscopic or histologic inflammation and these markers were found. Although mean CRP was minimally elevated above the established normal range for the dog (16–17.8 mg/L CRP) [37], 6 dogs had an abnormally high value. Normal synovial fluid CRP levels have not been reported for the dog and we did not identify a significant correlation with histologic or macroscopic assessment of synovitis. However, concentrations of CRP in stifle synovial fluid correlated with serum CRP. TNFα and INFγ may be too labile or secreted at too low of concentrations for detection using ELISA.

Survival analysis revealed that arthroscopic vascularity in the index stifle was the best predictor of subsequent contralateral CR. Additionally, increasing age was associated with reduced risk, presumably because the proportion of dogs without contralateral CR gradually decreases over time. Assessment of the vascularity in the index stifle may be a useful marker in longitudinal studies evaluating novel disease-modifying therapy that could modify the risk of subsequent contralateral rupture. In the longitudinal component of this study, we found that 52% of the cohort developed subsequent contralateral rupture with a median time to rupture of 593 days. These results are similar to other work [5],[23],[38]–[42], and suggest that the fiber fraying observed arthroscopically in the contralateral stable stifles [4] does indeed represent the incipient phase of the CR condition in which progressive ligament fraying eventually leads to development of stifle instability. The hypothesis that synovial inflammation within the stifle is a significant risk for progressive fiber tearing within the cruciate ligament complex over time is also supported by the observation that severity of radiographic synovial effusion in the contralateral stifle increases risk of subsequent contralateral CR [6],[38]. Risk factors for initiation of CR may not be the same as factors that promote disease progression. The present study does not advance mechanistic understanding of risk factors for disease initiation, which remains poorly understood [43],[44].

Several limitations were associated with this study. Histochemical or immunohistochemical staining for additional proteins may have improved our assessment of synovial inflammation. Correlations between arthroscopic and histologic data may have been more robust with an objective measurement for plasma cells and B lymphocytes, such as CD79a [45],[46], since plasma cells are an important component of the synovial inflammatory cell population in CR-affected dogs. Quantification of the level of expression of INFγ and TNFα at the mRNA level may have also improved assessment of effector cytokines important for T lymphocyte and macrophage signaling. Alternative handling of synovial biopsies, such as flash freezing immediately after collection, may have improved preservation of labile cytokines. Mini-arthrotomy was used for this study, rather than portal arthroscopy, principally to facilitate collection of synovial biopsies for histologic analysis without the use of a motorized shaver and to minimize the risk of any confounding effects on the arthroscopic appearance of the synovium. Extensive manipulation and or shaving of the soft tissues of the joint could also influence the appearance of the synovium or potentially remove synovial tissue of interest. Despite this approach, it is still possible that the biopsies we collected may not have fully captured regional variation in synovitis within the stifle. CD3^+^ T lymphocytes and TRAP^+^ macrophages, in particular, had a multifocal distribution within the synovium, making comprehensive assessment of the aggregates of cells within the synovium more difficult. Although development of subsequent contralateral rupture is typically associated with obvious lameness exacerbation, long-term follow-up for survival analysis was obtained by telephone in 8 of 27 dogs. Precision of case coding may have been improved if repeat patient examination had been performed in all dogs. Finally, inclusion of a larger number of dogs in the study may have improved interpretation of our results, particularly for survival analysis factors, such as neutering.

In conclusion, arthroscopic grading of synovitis is a valuable diagnostic tool that is significantly correlated with objective histologic markers of synovial inflammation with sufficient precision for use in longitudinal clinical studies. By using arthroscopy to better understand synovial pathology, a patient's status and response to treatment can be better understood. Significant correlations between arthroscopic hypertrophy, vascularity, and synovitis and synovial microarchitecture, including synovial intimal thickness, vasculature density, and numbers of T-lymphocytes were found. Stifle instability in dogs with CR is preceded by stifle synovitis that is associated with lameness, stifle joint OA, and fiber fraying in the cruciate ligament complex in dogs. Arthroscopic assessment of stifle synovitis could be used to help guide management decisions for dogs affected with incipient CR in a clinically stable stifle. Stifle synovitis is an early marker for the cruciate rupture arthropathy that could be therapeutic target for future longitudinal clinical trials evaluating disease-modifying treatment for the arthropathy in affected dogs as it is a significant risk factor for disease progression.

## Supporting Information

Appendix S1 **Canine stifle arthroscopic evaluation form.**
(DOCX)
