# Supplementary material for: Arthroscopic Assessment of Stifle Synovitis in Dogs with Cranial Cruciate Ligament Rupture
Source: PLoS One. 2014 Jun 3;9(6):e97329. doi: 10.1371/journal.pone.0097329 (PMC4043664; doi:10.1371/journal.pone.0097329)
Supplement: Appendix S1 — Canine stifle arthroscopic evaluation form. (DOCX) [file pone.0097329.s001.docx]

| **Canine Stifle Arthroscopic Evaluation Form**  **Date** _____________________  **DVM** _____________________  **Medical Record #** ___________  **Stifle** *Right Left*  **Cranial cruciate ligament Caudal cruciate ligament Meniscus**  Tear: *Normal Partial Full %* Tear: *Normal Partial* Medial: ______________  Lateral: ______________  **MACRO Score**^27^  **Hypertrophy:** (0) Represents a thin and transparent synovial membrane. Vessels are clearly visible, as is subsynovial tissue (e.g. fibrous capsule, fat or muscle). A score of 1 represents minor thickening, granulations score higher and club-like villi earn a maximum score (4). White fibrotic villi score the same as active hypervascularized villi.  **Vascularity:** Thin and scattered vessels score 0. Increasing vascularity is graded by increasing scores. Densely packed vessels score 4, and intermediate in between. Hyperaemia is not included in this parameter.  **Synovitis:** This parameter grades every visible aspect of synovitis as assessed globally by the observer. A higher score represents a higher degree of inflammation. White fibrotic tissue is scored 0, as is normal synovial membrane. Hyperaemia, which is not included in the vascularity index, is included here.   \| **Location** \| **Hypertrophy (0-4)** \| **Vascularity (0-4)** \| **Synovitis (0-4)** \| \| --- \| --- \| --- \| --- \| \| Lateral Pouch \|  \|  \|  \| \| Lateral Femoro-tibial \|  \|  \|  \| \| Medial Pouch \|  \|  \|  \| \| Medial Femoro-tibial \|  \|  \|  \| \| Intercondylar notch \|  \|  \|  \| \| Femoro-patellar \|  \|  \|  \| |
| --- | --- | --- | --- | --- | --- | --- | --- | --- | --- | --- | --- | --- | --- | --- | --- | --- | --- | --- | --- | --- | --- | --- | --- | --- | --- | --- | --- | --- |
